# Limb-Girdle Type Congenital Myasthenic Syndrome Anticipated With Scoliosis: A Case Report

**DOI:** 10.7759/cureus.71874

**Published:** 2024-10-19

**Authors:** Luka Kvirtia, Salome Gagnidze, Nana Tatishvili, Mariam Lomsianidze

**Affiliations:** 1 Medicine, David Tvildiani Medical University, Tbilisi, GEO; 2 Pediatric Neurology, M. Iashvili Children's Central Hospital, Tbilisi, GEO

**Keywords:** cms type 12, congenital myasthenic syndrome (cms), dysphagia, gfpt1 gene, limb-girdle type muscle weakness, respiratory insufficiency, scoliosis

## Abstract

Congenital myasthenic syndromes (CMS) comprise a group of inherited disorders that impair signal transduction as well as the structural integrity of the neuromuscular junction. Since there are several gene mutations associated with CMS, including the rare GFPT1 gene, the clinical presentation of this condition is quite variable. Importantly, patients with the same genotype can exhibit different clinical features. CMS type 12, as seen in our patient, is characterized by weakness of the limb-girdle muscles, which was anticipated with scoliosis. It is important to note that scoliosis is not generally linked to the GFPT1 mutation, and based on our case, it contributed to a broader and more complex clinical presentation.

## Introduction

Congenital myasthenic syndromes (CMS) are a group of inherited conditions that disrupt the neuromuscular junction (NMJ). The vast majority of these disorders are autosomal recessive, except dominant slow channel syndrome and CMS associated with SYT2. The NMJ comprises the presynaptic membrane of the motor neuron, the synaptic cleft, and the postsynaptic membrane of skeletal muscle fiber. The release of neurotransmitters from the presynaptic neuron and their subsequent attachment to postsynaptic membrane receptors constitute the complex process of synaptic transmission, which takes place at the NMJ. Many of our genes encode presynaptic, synaptic, and postsynaptic proteins that are essential in signal transmission at the NMJ. More than 30 gene mutations have been linked to the development of CMS [1,2].

In addition, there are also mutations in genes that influence the posttranslational modification of proteins critical for neuromuscular transmission. One prominent example is the GFPT1 mutation, which demonstrates its importance in this complex process. Glutamine-fructose-6-phosphate transaminase 1, encoded by GFPT1, is the first and rate-limiting enzyme in the pathway for the synthesis of hexosamines. The final product of this pathway is uridine diphosphate-N-acetylglucosamine (UDP-GlcNAc), which is crucial for both O- and N-glycosylation and functions as a universal precursor for all amino sugars required in the synthesis of glycoproteins, glycolipids, and proteoglycans. There are different types of proteins in the NMJ that are highly glycosylated, such as muscle-specific kinase (MuSK) and acetylcholine receptor (AChR) subunits. Therefore, mutations that affect glycosylation can disrupt neuromuscular transmission [1,2].

GFPT1 has a muscle-specific isoform, GFPTL1. Mutations resulting in this isoform are rare; the phenotype is usually severe and includes autophagic myopathy and significant dysfunction of the neuromuscular junction [3,4]. GFPT1 can, however, have mutations anywhere within its coding sequence; most of them are missense mutations, with few resulting in truncation of the protein [2].

GFPT1 mutation leads to CMS, which manifests as weakness of limb-girdle muscles; it progresses slowly and fluctuates in symptom severity. Usually, the symptoms of CMS type 12 appear between birth and the first two years of life, but patients can present much later [2]. Several similar clinical symptoms are typically shared by affected individuals, such as a marked and fatigable weakness of the proximal limb muscles, but ocular, facial, bulbar, or respiratory muscles are not generally involved [1]. Electrophysiological investigations include single fiber electromyography (SFEMG) and repetitive nerve stimulation (RNS) and show characteristics of postsynaptic involvement. The muscle biopsies obtained from CMS type 12 patients demonstrate tubular aggregates. Individuals with the condition typically benefit from acetylcholinesterase inhibitors [2]. In contrast, CMS caused by mutations in Dok7, AGRN, MuSK, and LRP4 can result in undesired effects from these drugs. However, they often respond positively to treatment with ephedrine or salbutamol [5].

In this case report, we present a patient with CMS type 12 manifested with atypical features, such as scoliosis, respiratory insufficiency, and bulbar muscle weakness.

## Case presentation

A 13-year-old patient was born via cesarean delivery at 41 weeks of gestation to nonconsanguineous parents. She developed respiratory distress syndrome after birth and was hospitalized for 25 days, although mechanical ventilation was not required, and she was discharged. During infancy, the patient exhibited muscle weakness that started almost equally in the upper and lower limbs but was slightly marked in the lower extremities.

Initially, the patient developed normally and was able to sit independently at seven months of age. By one year she could walk; however, she has always struggled with walking as she would tire easily. Fine motor milestones were normal. At 14 months, the patient experienced marked motor regression. She was unable to sit independently without assistance, also had difficulty with head control, and was unable to hold her head up independently; this led to the decision to undergo a rehabilitation course. Although it was possible to climb the stairs, running was always a hard task for her, and she also experienced frequent falls. The patient’s gait was unstable, and by the age of three, she was dependent on her mother to take steps. From the age of six, the patient was no longer able to walk, and by the age of eight, she could not stand on her own. During this time, she became wheelchair-bound. Jumping and squatting were never possible for her to perform. She can use her upper extremities functionally and can write well but is unable to lift her arms. There was no prior history of diplopia, eyelid drooping, limb numbness, muscle twitches, and no family history of a similar condition.

The patient developed severe scoliosis; the vertebral column was deformed, and at the level of the thoracolumbar spine, the deformation angle was 63 degrees. There was marked shoulder and hip asymmetry. The patient was unable to roll over and could only sit with support using hands or other assistive objects. At the age of 10, she experienced respiratory function impairment, and spirometry was indicated. Results revealed the restrictive type of ventilation defect, including a vital capacity (VC) of 47%, a forced expiratory volume (FEV1) of 50%, a forced vital capacity (FVC) of 40%, a peak expiratory flow (PEF) of 48%, and a forced expiratory time (FET) of 2.0 seconds (normal: 6 seconds).

The patient's examination revealed muscle atrophy at the shoulder girdle. There were contractures on both elbow joints as well as feet, especially marked on the left side. She had difficulty raising her arms and brushing her hair. In addition, muscle strength was more pronounced in her forearms than in her arms. These findings indicated proximal muscle weakness, which was more observable after exertion. Examination showed positive reflexes in the upper extremities, but knee and ankle reflexes were absent. She was alert and focused; the cranial nerve exam revealed no abnormalities. No meningeal, cerebellar, or sensory symptoms were present. Routine blood investigations showed normal results, although creatine kinase (CK) levels were elevated to 227 U/L (reference range: 34-145 U/L).

At the age of 12, genetic analysis was ordered, and a homozygous mutation was identified in the GFPT1 gene, which established the diagnosis of autosomal recessive congenital myasthenic syndrome type 12.

**Figure 1:**
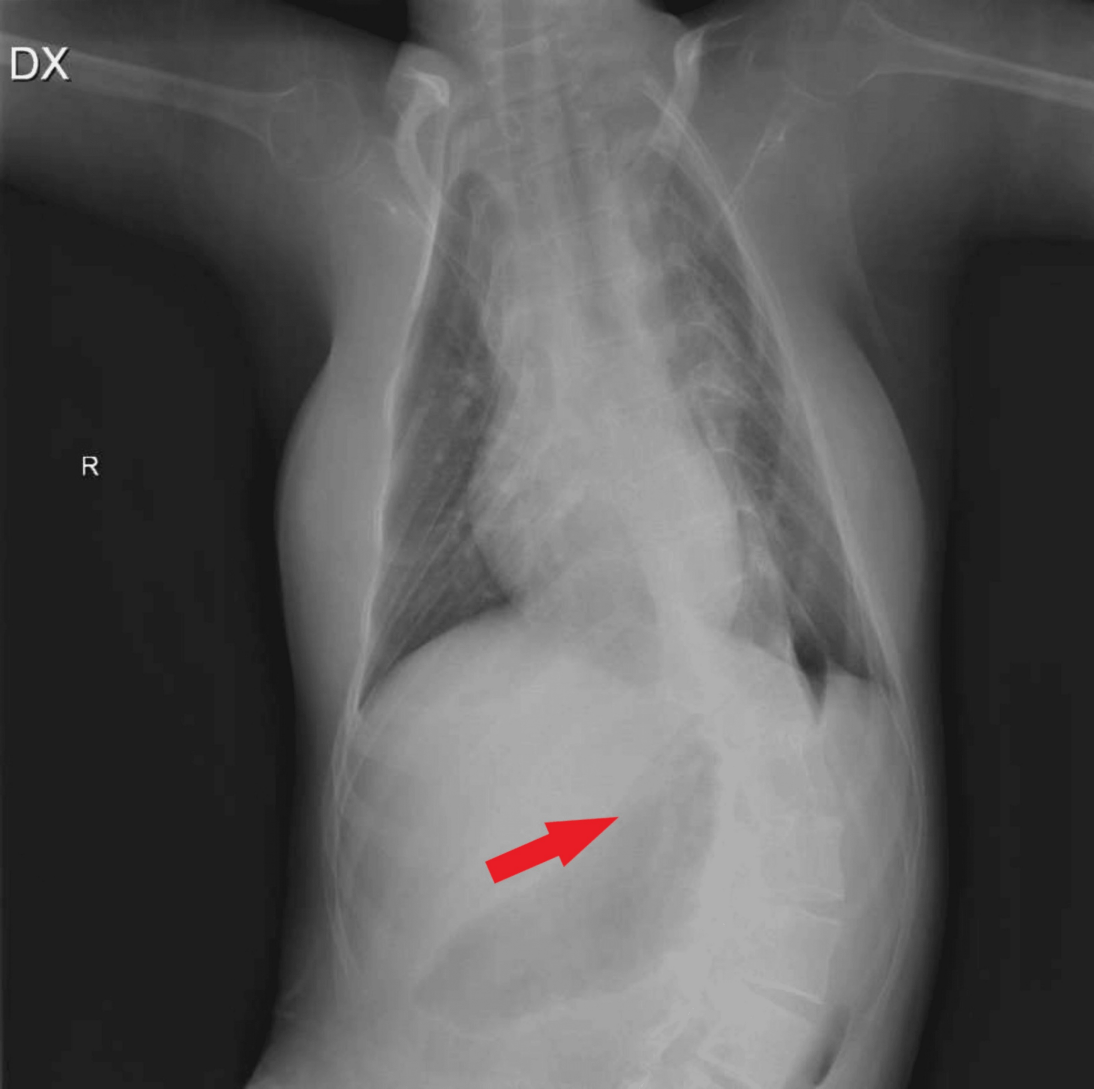
Preoperative chest X-ray of the patient shows severe scoliosis.

**Figure 2:**
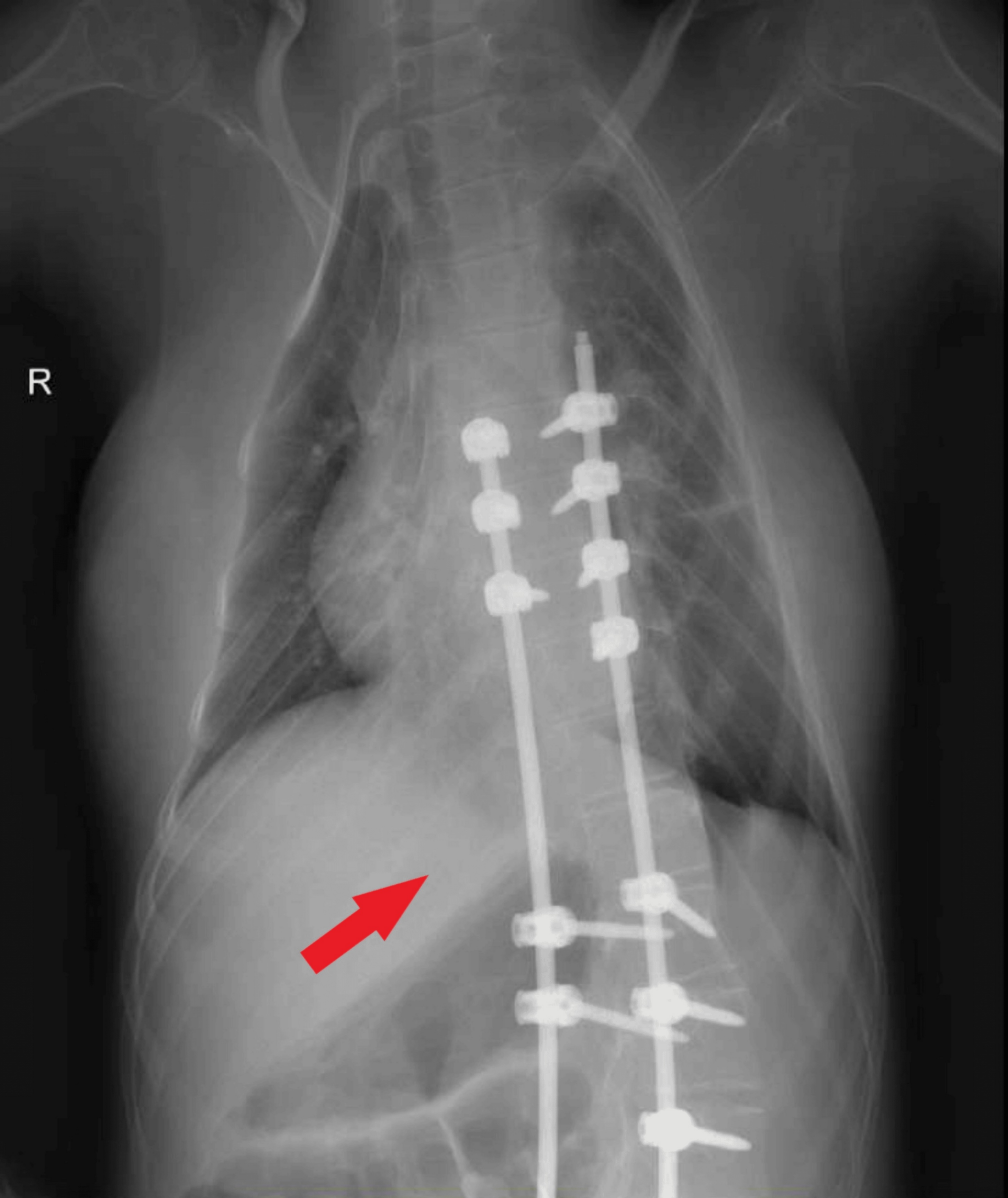
Postoperative chest X-ray of the patient shows reduction of the deformity.

Around this time, scoliosis progressed to an extent that it further deteriorated her respiratory function, and simultaneously she suffered from frequent infections that led to severe respiratory failure (Figure 1). As a result, the patient was transferred to the intensive care unit. Additionally, she was no longer able to sit and was bed-bound. Chewing and swallowing were also affected, which increased the time for food processing. The impairments mentioned above indicated the need for surgical intervention for scoliosis, and by the age of 13, surgery was performed with beneficial outcomes (Figure 2). The deformation angle is currently 34 degrees, and respiratory function, as well as swallowing and sitting, has improved. Although she now sits more stably, her walking and standing abilities are still unattainable. Furthermore, she can cough more effectively, and respiratory infections are not as severe as before.

Cognitive, language, and speech developmental milestones were consistent with her age on routine pediatric checkups. She has never experienced visual impairment. The patient was able to attend kindergarten and is currently performing well in school. She is quite emotional and experiences occasional breathing difficulties. One such episode was considered to be a panic attack. During attacks, her emotional state exacerbates the restrictive pattern of ventilation, making her unable to breathe freely.

At the time of diagnosis, the patient started Mestinon 60 mg three times a day. A few months after the initiation of treatment, spirometry was performed again, showing improved results; however, a restrictive pattern of ventilation was still present: VC of 59%, FEV1 of 62%, FVC of 70%, PEF of 57%, FEV1/FVC of 92%, and FET of 5.7 seconds (normal: 6 seconds). Soon after the surgery, the patient had marked improvement in the condition as mentioned above.

As for today, she manages to roll over slowly and independently but mainly relies on her mother for assistance with daily activities.

## Discussion

In this publication, we describe the first reported case of CMS type 12 from Georgia. Due to a lack of awareness of the GFPT1 mutation, we think it’s crucial to present the detailed course of the condition to decrease the number of undiagnosed cases. Our patient presented with limb-girdle-type weakness while sparing the ocular and facial muscles, with no ptosis or diplopia, which is characteristic of CMS type 12. Since she had pronounced proximal muscle weakness, fine motor skills, including writing and drawing, developed without delay. Moreover, there was marked atrophy of shoulder girdle muscles. It should be mentioned that CK levels were elevated in our patient, which is observed in only one-fourth of individuals with GFPT1 mutation [6]. She exhibits contractures in both elbows and feet that are not typically associated with the mutation [7]. Although the disease course is benign and progresses slowly [6], her condition worsened remarkably early in life and experienced marked motor regress, including the inability to walk or stand, which caused her to be dependent on a wheelchair.

As we have already mentioned above, the patient had respiratory insufficiency, which is not commonly seen in the GFPT1 mutation; it is usually associated with mutations of RAPSYN, COLQ, MUSK, and others [7]. This complication may arise from scoliosis, respiratory muscle weakness, or a combination of both. The development of respiratory insufficiency was also significantly influenced by recurrent infections. Preventive immunizations were therefore suggested to reduce further infections. It is reasonable to consider scoliosis as a possible cause of respiratory insufficiency in our patient since severe cases might impair respiratory mechanics and result in restrictive lung disease. The fact that the respiratory condition was improved after the surgical intervention supports the outcomes of scoliosis mentioned above. However, prior to surgery, Mestinon was initiated, and spirometry revealed improved ventilation, which may indicate a more complex underlying mechanism. Although these considerations provide some understanding, given that the GFPT1 mutation is not often associated with respiratory difficulties, they do not entirely explain the observed problem and warrant further consideration. Furthermore, the patient exhibited dysphagia, which likely stemmed from the weakness of bulbar muscles, which is not generally seen in CMS type 12. We also cannot exclude scoliosis as a potential cause of swallowing difficulty since it improved following surgical intervention. Finally, scoliosis itself is typically associated with mutations of the COLQ, VAMP1, and CHRNE (slow channel syndrome) genes rather than GFPT1 mutations [7].

In rare cases, the GFPT1 mutation is associated with a certain level of cognitive impairment that ranges from mild learning difficulties to autism spectrum disorder [2]. However, our patient never had such issues; as we have already mentioned, her academic performance was always satisfactory.

Since CMS is not a common disorder and its clinical presentation can be similar to other conditions, the diagnosing process in this case was challenging. Furthermore, because genetic analysis is typically performed outside of Georgia, it took longer to establish a definitive diagnosis. One of the important differential diagnoses was limb-girdle muscular dystrophy (LGMD) due to its early onset, atrophy of the shoulder girdle muscles, normal intellectual development, absence of distal muscle weakness, and increased CK [8]. The overlap of symptoms, as well as elevated CK levels, initially suggested LGMD, which indicated further genetic analysis to ultimately establish the diagnosis. Following the identification of the GFPT1 mutation, additional antibody tests are typically done for CMS (antibodies directed against AChR, MuSK, and the voltage-gated P/Q type calcium channel) [6], and RNS was not performed.

An additional differential diagnosis to consider was spinal muscular atrophy (SMA) type two, which is associated with motor skill impairments, hypotonia, lack of intellectual disability, feeding difficulties, a weak cough, joint contractures, and scoliosis [9]. Eventually, whole exome sequencing allowed us to exclude all the other potential disorders, confirming the mutation of GFPT1 and diagnosis of CMS type 12.

## Conclusions

To conclude, congenital myasthenic syndrome is a rare genetically inherited disorder, especially caused by the GFPT1 mutation. Since it’s quite uncommon and its clinical picture overlaps with other conditions, CMS type 12 often goes undiagnosed. Genetic analysis is the method of choice to establish the diagnosis, and it is the only way to exclude all other conditions similar to CMS. Given the limited number of case reports about the GFPT1 mutation currently available, it is critical to report our patient’s case to provide new associations and increase awareness of the condition, which will aid in early recognition and facilitate the prompt initiation of appropriate treatment.
